# Chinese herbal medicine for the treatment of small intestinal bacterial overgrowth (SIBO): A protocol for systematic review and meta-analysis

**DOI:** 10.1097/MD.0000000000023737

**Published:** 2020-12-18

**Authors:** Xuetong Ren, Zirui Di, Zhe Zhang, Bingqian Fu, Yuman Wang, Chongxin Huang, Yanru Du

**Affiliations:** a Hebei University of Chinese Medicine; b The First Affiliated Hospital of Hebei University of Chinese Medicine, Shijiazhuang City, Hebei, China.

**Keywords:** Chinese herbal medicine, small intestinal bacterial overgrowth, protocol, systematic review

## Abstract

**Background:**

Chinese medicine has a unique theory and the Chinese herbal medicine treatment is based on the integral concepts and syndrome differentiation of the Traditional Chinese Medicine system. Although antibiotics remain the mainstay of SIBO treatment, various alternative or adjunctive therapies are available, including prokinetic agents, dietary interventions, probiotics, and herbal combinations. There is accumulating evidence demonstrating the antimicrobial properties of a growing number of herbs including garlic, black cumin, cloves, cinnamon, thyme, all-spices, bay leaves, mustard, and rosemary. This has prompted an interest in herbal therapy for the treatment of SIBO. Currently, there is no systematic review focusing on efficacy of CHM in the treatment of SIBO with PCOS, so our meta-analysis aims to comprehensively explore it. Meanwhile we will provide high-quality evidence to help patients, clinicians as well as health policymakers select better treatment strategy of PCOS.

**Methods:**

We will search the following sources without restrictions for date, language, or publication status: PubMed, Web of Science, Cochrane Central Register of Controlled Trials (CENTRAL) Cochrane Library, EMBASE and China National Knowledge Infrastructure. We will apply a combination of Medical Subject Heading (MeSH) and free-text terms incorporating database-specific controlled vocabularies and text words to implement search strategies. We will also search the ongoing trials registered in the World Health Organization's International Clinical Trials Registry Platform. Besides, the previous relevant reviews conducted on CHM for SIBO and reference lists of included studies will also be searched.

**Results:**

This study will provide a reliable basis for the treatment of SIBO with CHM.

**Conclusions:**

The findings will be an available reference to evaluate the efficacy and safety of CHM in the treatment of SIBO.

**Registration number:**

INPLASY202080004.

## 1 Introduction

Small intestinal bacterial overgrowth (SIBO) is a heterogeneous syndrome characterised by an increased number and/or abnormal type of bacteria in the small bowel, and it is a well-recognised cause of maldigestion and malabsorption. SIBO is defined as an overgrowth of intestinal bacteria proximal to the colon that can lead to a wide variety of clinical manifestations ranging from completely asymptomatic to malabsorption, steatorrhea, and weight loss in severely affected cases.^[1–4]^ Most often, patients present with nonspecific symptoms including bloating, flatulence, abdominal pain, diarrhea, and constipation. Unlike the colon, the human small intestine is typically an inhospitable environment for bacteria to grow and flourish. This is due to a variety of host factors, which include normal fasting and fed motility, secretion of gastric acid, pancreaticobiliary secretions, structural barriers such as the ileocecal valve, an intact gut immune system, and commensal bacteria. Situations that disrupt this natural environment can lead to gut dysbiosis, as well as SIBO.^[5–8]^ When these protective mechanisms are compromised, bacteria are able to populate the small bowel.^[9]^

SIBO has been recognized as a medical phenomenon for many decades. Risk factors for developing SIBO include anatomic abnormalities, hypochlorhydria, motility disorders, organ system dysfunction, age, medications, and pre-existing conditions such as irritable bowel syndrome, celiac disease, and nonalcoholic steatohepatitis.^[10]^ Small bowel culture is widely accepted as the “best diagnostic method” for establishing a diagnosis of SIBO; a threshold of ≥103 cfu/mL is recommended as a positive test result for SIBO, especially when performing duodenal aspirate and culture, because of very low bacterial counts in this more acidic environment.^[11–13]^ Breath testing is a safe and noninvasive diagnostic method for SIBO. However, there is currently no standard methodology for breath testing. The use of antibiotics has been the cornerstone of therapy for the treatment of SIBO. Indeed, based solely on anecdotal evidence, it has been a longstanding common practice to use empiricantibiotic therapy in those with risk factors for and a clinical presentation suggestive of SIBO.

Since the late 1990s, there has been a resurgence in SIBO research which has been further enhanced by the increasing knowledge of the gut microbiome and its roles in human health and disease. These include a series of articles linking SIBO to diseases such as irritable bowel syndrome, inflammatory bowel disease, systemic sclerosis, motility disorders, cirrhosis, fatty liver, postgastrectomy syndrome, and a variety of other conditions. Although these findings are important, a recent consensus document identified a number of strengths and weaknesses in the published work in this area. As such, an effort has been underway to re-evaluate the criteria for the diagnosis of SIBO and define the optimal methods for diagnostic testing to identify this condition. Furthermore, treatment for SIBO has been largely empirical, has not undergone the scrutiny of sponsored clinical trials, and requires appraisal.^[12,14–19]^

Chinese medicine has a unique theory and the Chinese herbal medicine treatment is based on the integral concepts and syndrome differentiation of the Traditional Chinese Medicine (TCM) system.^[20]^ Although antibiotics remain the mainstay of SIBO treatment, various alternative or adjunctive therapies are available, including prokinetic agents, dietary interventions, probiotics, and herbal combinations. There is accumulating evidence demonstrating the antimicrobial properties of a growing number of herbs including garlic, black cumin, cloves, cinnamon, thyme, all-spices, bay leaves, mustard, and rosemary. This has prompted an interest in herbal therapy for the treatment of SIBO.^[21,22]^ Currently, there is no systematic review focusing on efficacy of CHM in the treatment of SIBO with PCOS, so our meta-analysis aims to comprehensively explore it. Meanwhile we will provide high-quality evidence to help patients, clinicians as well as health policymakers select better treatment strategy of PCOS.

## 2 Objective

The objective of this systematic review is to identify, analyse, and synthesize research evidence on the effectiveness and safety of CHM in the treatment of SIBO.

## 3 Methods and analysis

### 3.1 Study registering and reporting

We have registered this study on INPLASY202080004. This systematic review protocol will be prepared to underlie the Preferred Reporting Items for Systematic Review and Meta-Analysis Protocols (PRISMA-P) guidance. We will record any protocol changes made during the implementation of the review in the publication of the final report. The PRISMA extension declaration is a declaration that ensures that all aspects of the method and result are reported. We followed the PRISMA-P guidelines.^[23]^

### 3.2 Eligibility criteria

The design of inclusion criteria and exclusion criteria in this study is based on the 5 main principles of PICOS.

#### 3.2.1 Type of Participants

Inclusion: Adults with SIBO (as diagnosed using any recognised diagnostic criteria).

Exclusion: Adolescents (under 18 years of age) and elderly people (over 70).

#### 3.2.2 Type of Interventions and comparators

Intervention: Use traditional Chinese medical herbal treatment.

Comparator: The control group included placebo, no treatment, and western medicine.

#### 3.2.3 Outcomes

The Main outcomes are the efficacy and safety of CHM in the treatment of SIBO. And the additional outcomes included: Determination of methane hydrogen breath test, hemoglobin, folic acid, vitamin B12 content and gastronic acid function.

#### 3.2.4 Study design

In order to limit heterogeneity and enhance clinical applicability, strict inclusion/exclusion criteria were established. Only the RCTS were included for analysis. We will rule out repeated studies that do not have enough information to calculate effect estimates. We will not apply any language or other restrictions.

### 3.3 Information source

We will search the following sources without restrictions for date, language, or publication status: PubMed, Web of Science, Cochrane Central Register of Controlled Trials (CENTRAL) Cochrane Library, EMBASE and China National Knowledge Infrastructure. We will apply a combination of Medical Subject Heading (MeSH) and free-text terms incorporating database-specific controlled vocabularies and text words to implement search strategies. We will also search the ongoing trials registered in the World Health Organization's International Clinical Trials Registry Platform. Besides, the previous relevant reviews conducted on CHM for SIBO and reference lists of included studies will also be searched.

### 3.4 Search strategy

Two authors will screen the titles and abstracts of the all records retrieved in above electronic databases independently to find potentially eligible reviews. According to the inclusion and exclusion criteria outlined above, the full texts of them will be retrieved for further identification. Any disagreement will be resolved by discussion or by consultation with a third author. The search strategy for PubMed is presented in Table 1 and the strategy will be modified upon the requirement of other databases.

**Table 1:** Search strategy used in PubMed database.

Number	Search terms
#1	Small intestinal bacterial overgrowth[Title/Abstract] OR SIBO[Title/Abstract] OR Enteric bacterial overgrowth syndrome [Title/Abstract]
#2	Traditional Chinese Medicine[MeSH Terms]
#3	Chung I Hsueh[Title/Abstract] OR Hsueh, Chung I[Title/Abstract] OR Traditional Medicine, Chinese[Title/Abstract] OR Zhong Yi Xue[Title/Abstract] OR Chinese Traditional Medicine[Title/Abstract] OR Chinese Medicine, Traditional[Title/Abstract] OR Traditional Tongue Diagnosis[Title/Abstract] OR Tongue Diagnoses, Traditional[Title/Abstract] OR Tongue Diagnosis, Traditional[Title/Abstract] OR Traditional Tongue Diagnoses[Title/Abstract] OR Traditional Tongue Assessment[Title/Abstract] OR Tongue Assessment, Traditional[Title/Abstract] OR Traditional Tongue Assessments[Title/Abstract]
#4	#2 OR #3
#5	randomized controlled trial[Publication Type]
#6	randomized[Title/Abstract]
#7	randomly[Title/Abstract]
#8	#5 OR #6 OR #7
#14	#1 AND #4 AND #8

### 3.5 Data collection and analysis

#### 3.5.1 Study selection

Two reviewers will perform literature screening, study selection, and data extraction independently. The literature obtained will be imported into EndnoteX9 to screen the title and abstract, the duplications and studies failing to meet the pre-specified inclusion criteria will be excluded. After reading the full text of the remained literature and discussing within the group, the final included studies will be determined. The corresponding author of original RCT will be contacted when the full text is unavailable. Disagreements will be solved by consulting a third-party arbitrator or discussing within a group.

#### 3.5.2 Data extraction and management

Two authors will screen the titles and abstracts of the all records retrieved in above electronic databases independently to find potentially eligible reviews. According to the inclusion and exclusion criteria outlined above, the full texts of them will be retrieved for further identification. Any disagreement will be resolved by discussion or by consultation with a third author.

Data will be extracted by two reviewers independently using a pre-designed data extraction form. A third reviewer will validate data. The following data will be extracted: General information, Trial characteristics, Intervention(s) and control(s), Participants, Study methodology, Outcomes, Results, etc.

#### 3.5.3 Risk of bias in included studies

The methodological quality of eligible studies will be assessed by two review authors independently according to the the Cochrane Handbook for Systematic Reviews of Interventions. The following characteristics will be assessed: random sequence generation (selection bias), allocation concealment (selection bias), blinding of participants and personnel (performance bias), blinding of outcome assessment (detection bias), incomplete outcome data (attrition bias), selective reporting (reporting bias), other bias. Based on the assessments of the studies against these seven domains, they will be classified as being of “low risk”, “high risk” or “unclear risk” of bias. Any disagreements will be resolved by discussion or discussed with another reviewer if necessary.

#### 3.5.4 Strategy of data synthesis

Meta-analysis was conducted using Review Manager software (version 5.3). Odds ratio (OR) with 95% confidence intervals (CI) was reported for the dichotomous data, and mean differences (MD) with 95% CI for the continuous data. Statistical heterogeneity between studies was tested by calculating Higgins I^2^ values or using the χ^2^ test. I^2^ > 25%, I^2^ > 50%, and I^2^ > 75% were respectively defined to indicate moderate, substantial, and considerable heterogeneity. When the *P* value of χ^2^ test was <.1, an I^2^ test was carried out. If the I^2^ test showed a value >50%, a random effects model was carried out. Otherwise, a fixed effects model was carried out. A *P* value lower than .05 was considered to be statistically significant.

#### 3.5.5 Subgroup analysis

If results of the meta analysis are significantly heterogeneous, subgroup analyses of the control groups might be performed.

#### 3.5.6 Sensibility analysis

If sufficient trials are identified, we plan to conduct a sensitivity analysis comparing the results using all trials with high methodological quality: studies classified as having a ‘low risk of bias’ versus those identified as having a ‘high risk of bias’.

#### 3.5.7 Patient and public involvement

This is a meta-analysis study based on previously published data, so patient and public involvement will not be included in this study.

#### 3.5.8 Grading the quality of evidence

The Grading of Recommendations Assessment, Development and Evaluation (GRADE) guidelines will be utilized to grade the quality of evidence as very low, low, moderate, or high.

## 4 Discussion

Small intestinal bacterial overgrowth is a clinical condition characterized by a malabsorption syndrome because of an increase in micro-organisms to a level exceeding 10^5^ bacteria/mL of jejunal juice.^[23]^ Antibiotic therapy is the cornerstone of the treatment of SIBO. In recent years, it has been reported that Chinese herbal medicine has a certain therapeutic effect on the overgrowth of small intestinal bacteria.^[1]^ Though there are several issues to be understood, CHM has been as an innovative approach to SIBO. However, as far as we know, no literature review systematically assesses the efficacy and safety of CHM in the treatment of SIBO. Therefore, this systematic review will investigate the efficacy and safety CHM in the treatment of SIBO. We expect that this study may provide a basis for CHM for the treatment of SIBO, and may provide better options for the treatments to such patients.

## Author contributions

**Conceptualization:** Du Yanru.

**Data curation:** Ren Xuetong.

**Formal analysis:** Di Zirui.

**Funding acquisition:** Du Yanru.

**Methodology:** Fu Bingqian.

**Software:** Zhang Zhe, Wang Yuman, Huang Chongxin.

**Supervision:** Du Yanru.

**Writing – original draft:** Ren Xuetong.
